# Cyanophage Diversity and Community Structure in Dead Zone Sediments

**DOI:** 10.1128/mSphere.00208-21

**Published:** 2021-04-28

**Authors:** Elias Broman, Karin Holmfeldt, Stefano Bonaglia, Per O. J. Hall, Francisco J. A. Nascimento

**Affiliations:** a Department of Ecology, Environment and Plant Sciences, Stockholm University, Stockholm, Sweden; b Baltic Sea Centre, Stockholm University, Stockholm, Sweden; c Centre for Ecology and Evolution in Microbial Model Systems (EEMiS), Linnaeus University, Kalmar, Sweden; d Department of Biology, University of Southern Denmark, Nordcee and HADAL, Odense, Denmark; e Department of Marine Sciences, University of Gothenburg, Gothenburg, Sweden; Aix-Marseille University

**Keywords:** DNA, anoxic, cyanobacteria, cyanophages, sediment, virus

## Abstract

Up to 20% of prokaryotic organisms in the oceans are estimated to die every day due to viral infection and lysis. Viruses can therefore alter microbial diversity, community structure, and biogeochemical processes driven by these organisms. Cyanophages are viruses that infect and lyse cyanobacterial cells, adding bioavailable carbon and nutrients into the environment. Cyanobacteria are photosynthesizing bacteria, with some species capable of N_2_ fixation, which are known to form large blooms as well as resistant resting cells known as akinetes. Here, we investigated cyanophage diversity and community structure plus cyanobacteria in dead zone sediments. We sampled surface sediments and sequenced DNA and RNA, along an oxygen gradient—representing oxic, hypoxic, and anoxic conditions—in one of the world’s largest dead zones located in the Baltic Sea. Cyanophages were detected at all stations and, based on partial genome contigs, had a higher alpha diversity and different beta diversity in the hypoxic-anoxic sediments, suggesting that cyanobacteria in dead zone sediments and/or environmental conditions select for specific cyanophages. Some of these cyanophages can infect cyanobacteria with potential consequences for gene expression related to their photosystem and phosphate regulation. Top cyanobacterial genera detected in the anoxic sediment included *Dolichospermum*/*Anabaena*, *Synechococcus*, and *Cyanobium*. RNA transcripts classified to cyanobacteria were associated with numerous pathways, including anaerobic carbon metabolism and N_2_ fixation. Cyanobacterial blooms are known to fuel oxygen-depleted ecosystems with phosphorus (so-called internal loading), and our cyanophage data indicate the potential for viral lysis of cyanobacteria which might explain the high nutrient turnover in these environments.

**IMPORTANCE** Cyanophages are viruses that target cyanobacteria and directly control their abundance via viral lysis. Cyanobacteria are known to cause large blooms in water bodies, substantially contributing to oxygen depletion in bottom waters resulting in areas called dead zones. Our knowledge of cyanophages in dead zones is very scarce, and so far, no studies have assembled partial cyanophage genomes and investigated their associated cyanobacteria in these dark and anoxic sediments. Here, we present the first study using DNA and RNA sequencing to investigate *in situ* diversity of cyanophages and cyanobacteria in dead zones. Our study shows that dead zone sediments contain different cyanophages compared to oxic sediments and suggest that these viruses are able to affect cyanobacterial photosystem and phosphate regulation. Furthermore, cyanophage-controlled lysis of cyanobacteria might also increase the turnover of carbon, phosphorus, and nitrogen in these oxygen-free environments at the bottom of the sea.

## Abstract (precis)

Cyanophages are viruses that target cyanobacteria and directly control their abundance via viral lysis. Cyanobacteria are known to cause large blooms in water bodies, substantially contributing to oxygen depletion in bottom waters resulting in areas called dead zones.

## INTRODUCTION

Viruses are up to 15-fold more abundant than prokaryotic organisms, and up to 20% of all microorganisms in the oceans are estimated to die daily because of viral infection and lysis (1). Viruses are therefore able to alter microbial diversity, community structure, and biogeochemical processes driven by these microorganisms (1). Among viruses with important ecosystem effects are cyanophages. These viruses are known to infect cyanobacteria, and after infection, lytic cyanophages eventually lyse the host cell (2–4) and thus contribute to the release of carbon and nutrients into the environment (5, 6). Many cyanobacterial species are capable of photosynthesis and N_2_ fixation (7) and are therefore essential primary producers channeling organic carbon and nitrogen to higher trophic levels in both aquatic and terrestrial ecosystems (8–12). While some cyanobacteria, such as the genus *Synechococcus*, have been shown to proliferate in oligotrophic waters (13), other genera, e.g., *Aphanizomenon*, *Nodularia*, and *Dolichospermum* (previously classified as planktonic *Anabaena* [14]), form large blooms in aquatic systems during high-nutrient-load—yet nitrogen-limited—and warm periods (15, 16). A portion of these blooms eventually decay and sink down below the euphotic zone and settle on the seabed (15, 17, 18). Cyanophages can increase the decay of these pelagic cyanobacteria while sinking down toward the seabed through increased particle formation caused by viral lysis (19). On the sediment surface, the settled cyanobacterial bloom material provides organic matter for benthic consumers and adds to the internal loading of phosphorus in the system (8, 12, 20, 21). Eventually, microbial degradation of this material and other sources of organic matter together with water stratification depletes oxygen in the bottom water, and so-called hypoxic (<63 μM O_2_) or anoxic “dead zones” are formed (17, 22). As a result of eutrophication, dead zones can today be found in coastal areas adjacent to all populated continents (23) and consist of habitats rich in organic carbon and ammonium. In addition to sinking decaying blooms, cyanobacteria can also sink to the sediment as resting cells called akinetes that tolerate harsh conditions (24). Cyanobacteria are also known to survive these dead zones despite darkness (25, 26) and anoxic conditions (27) and might therefore be infected by cyanophages in dead zone sediments.

One of the largest dead zones in the world is located in the Baltic Sea (23), which hosts large cyanobacterial blooms in the surface water during summer (28), and anoxic waters below a permanent oxycline situated at 65- to 125-m depth (29). Viruses have been shown to be active and persist in sediment for thousands of years (30, 31). For example, Cai et al. (31) showed that cyanophages persist for centuries in dead zones in the Baltic Sea, indicating that cyanophages are present in the sediment in addition to newly recruited phages associated with sinking cyanobacteria. Cyanobacterial blooms have been traced back to ca. 7,000 years ago in the Baltic Sea (based on pigment analysis in deep sediments) (32), and it is therefore likely that the ancient cyanophages are remnants from these sinking blooms. Considering that cyanophages lyse cyanobacterial cells that release bioavailable carbon and nutrients (5), cyanophages might have a key role in the food web of dead zone sediments. Even though it is known that cyanophages are present in dead zones, there is a knowledge gap regarding their diversity, community composition, and associated cyanobacteria.

Here, we aimed to elucidate the role of *in situ* cyanophages and cyanobacteria in dead zone sediments. To investigate this aim, we sampled the top 0- to 2-cm sediment surface from four stations along a 60- to 210-m water depth gradient in one of the largest dead zones in the world (the Baltic Sea). These stations included oxic (station A), hypoxic (D), near-anoxic (E), and anoxic (F) sediment (Table 1; oxygen data first reported in the work of Broman et al. [33]). In addition to the oxygen gradient, stations D and F have been reported to have higher concentrations of toxic hydrogen sulfide (H_2_S), and station E higher concentrations of nitrous oxide (N_2_O) (33). DNA and RNA were extracted and sequenced using the latest sequencing technology (Illumina NovaSeq) to investigate cyanophages and cyanobacteria. We hypothesized that (i) the diversity and community structure of cyanophages are different along the oxic-anoxic biogeochemical gradient and (ii) any such difference is linked to changes in cyanobacterial community composition and RNA transcripts.

**TABLE 1:** Sampling conditions^*a*^

Station	Water depth (m)	O_2_ (μmol liter^−1^)	Date	Longitude	Latitude
A	60	330	April 25	19°04′951	57°23′106
D	130	8.8	April 26	19°19′414	57°19′671
E	170	1.8	April 23	19°30′451	57°07′518
F	210	0	April 23	19°48′035	57°17′225

a Triplicate sediment samples (top 0- to 2-cm sediment surface) were collected during 2018 in the Eastern Gotland basin dead zone in the Baltic Sea. The stations were located along a water depth gradient (all aphotic) with different bottom water oxygen conditions. Oxygen concentrations shown were measured in the top 0- to 0.5-mm sediment surface, with station A being oxic, D hypoxic, E near-anoxic, and F anoxic. Oxygen data were first reported in the work of Broman et al. (33).

## RESULTS

### Cyanophage diversity and community composition.

**FIG 1:**
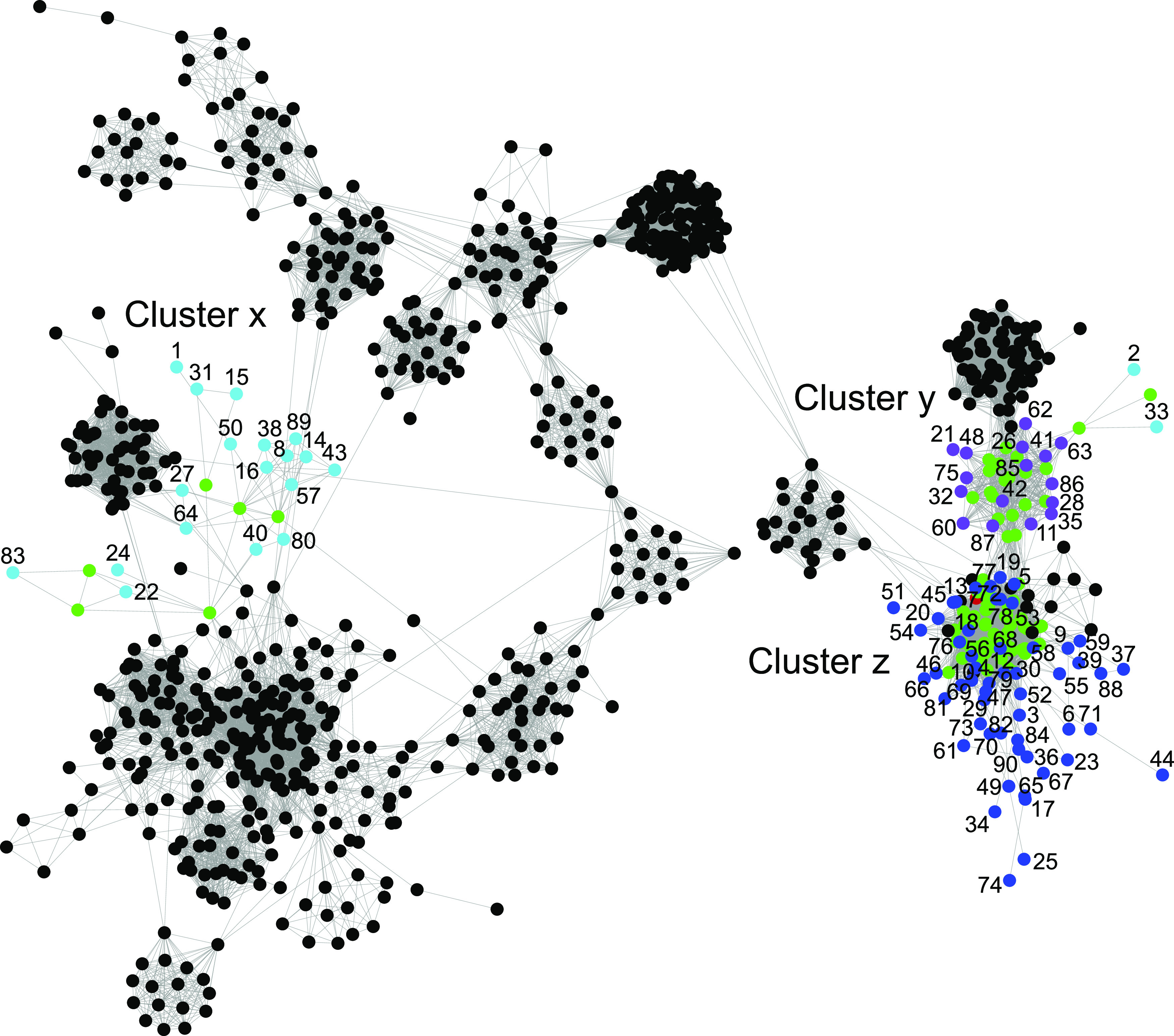
Network analysis consisting of reference viruses (NCBI bacterial and archaeal viral RefSeq V88) and the constructed virus contigs. The black nodes show noncyanophage viruses from the reference database, while green nodes show known cyanophages. The constructed contigs classified as viruses by VirSorter and associated with cyanophages in the vConTACT network analysis are denoted as turquoise, purple, and blue nodes (followed by the contig ID number) and clustered into three groups in the network (x, y, and z). Color codes: turquoise, cyanophages belonging to the family *Siphoviridae*; purple, *Podoviridae*; blue, *Myoviridae*; red, no taxonomic classification.

**FIG 2:**
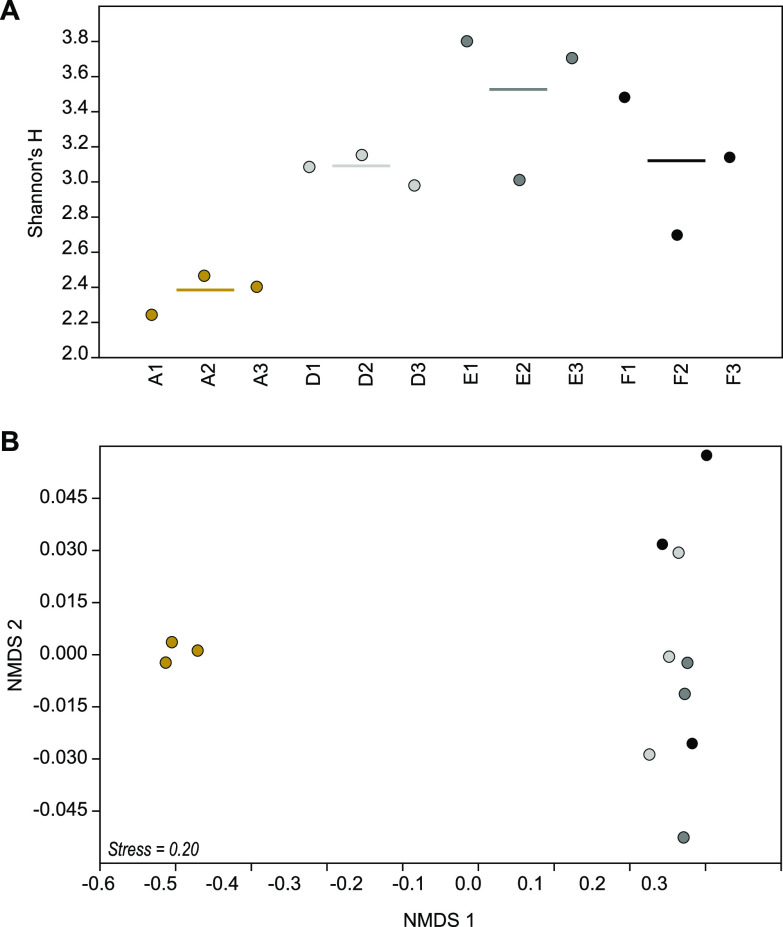
(A) Alpha diversity (Shannon’s H) of cyanophage contigs present in the samples (with at least 75% mapped read coverage). The bars show average values per station. (B) NMDS showing Bray-Curtis beta diversity of the cyanophages between the stations. The colors denote the different stations as follows: brown, A (oxic); light gray, D (hypoxic); dark gray, E (near-anoxic); and black, F (anoxic).

A total of 90 partial cyanophage genomes (i.e., contigs) were assembled from the coassembly metagenome data with an average genome size of 5,076 bp (range 2,000 to 47,157 bp). These partial cyanophage genome sequences were used in network analyses using vConTACT (34), which clusters genome-wide proteins based on the Markov Cluster Algorithm (MCL), and showed that the cyanophages formed three clusters (Fig. 1). These clusters were similar to *Siphoviridae* cyanophages (x), *Podoviridae* cyanophages (y), and *Myoviridae* cyanophages (z) (according to the cyanophage taxonomic classifications) (Fig. 1). See Fig. S1 in the supplemental material for the whole vConTACT network analysis including all reference viruses. Of these 90 identified cyanophages, 77 had a mapped read coverage of at least 75% and were therefore considered to be present in the sediment at the stations (Fig. 2 and see Data Set S1 for a full list of the cyanophages and their classified closest relative according to NCBI NT).

10.1128/mSphere.00208-21.1 FIG S1 Network showing all virus reference nodes (noncyanophages as black and cyanophages as green) alongside identified cyanophage contigs (blue nodes). Download FIG S1, PDF file, 1.3 MB. Copyright © 2021 Broman et al. 2021 Broman et al. https://creativecommons.org/licenses/by/4.0/ This content is distributed under the terms of the Creative Commons Attribution 4.0 International license.

10.1128/mSphere.00208-21.5 DATA SET S1 Constructed cyanophage contigs classified by first detecting virus contigs using VirSorter and then classified further as cyanophages based on protein content using vConTACT. The values in the table show normalized mapped bp values: bp mapped on contig/contig length (in kb)/metagenome bases (in Mb). The NCBI NT classifications of the contigs are also shown in the table. Download Data Set S1, XLSX file, 0.2 MB. Copyright © 2021 Broman et al. 2021 Broman et al. https://creativecommons.org/licenses/by/4.0/ This content is distributed under the terms of the Creative Commons Attribution 4.0 International license.

**FIG 3:**
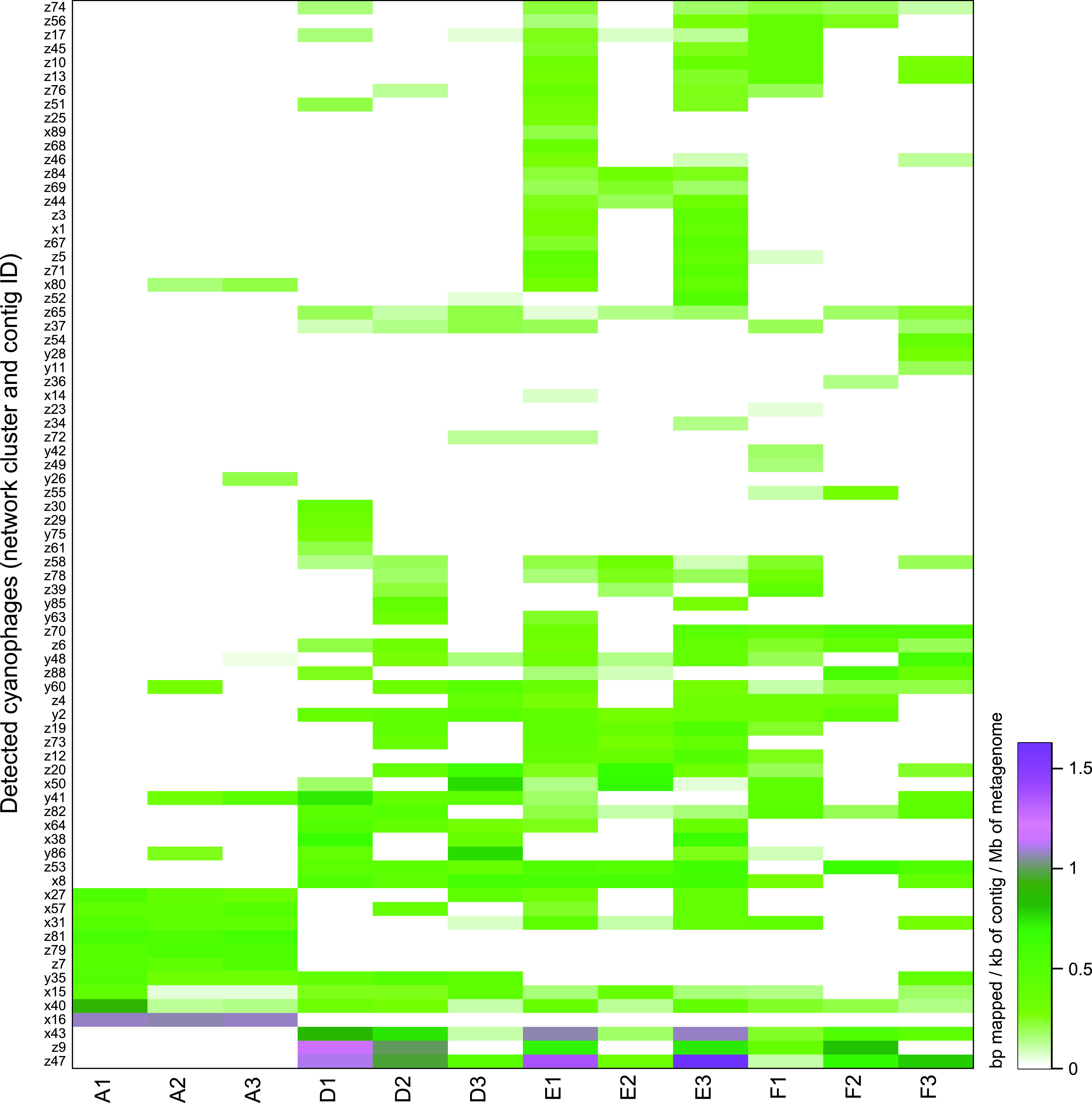
One-way clustered heatmap by rows showing cyanophage constructed contigs with a minimum of 75% coverage. The green-purple color gradient shows normalized sequence counts (mapped bp per contig/contig length in kb/metagenome in Mb). The row labels show contig IDs and their associated network cluster in Fig. 1 (x, y, or z).

Shannon’s H alpha diversity was higher for cyanophage contigs in the dead zone sediment, with: 3.07 ± 0.11 (station D, *P* = 0.081), 3.51 ± 0.43 (E, *P* = 0.007), and 3.11 ± 0.39 (F, *P* = 0.067) compared to 2.37 ± 0.11 at station A [*n* = 3 per station, 1 standard deviation shown, one-way analysis of variance (ANOVA) with Tukey *post hoc* test, whole model *F*_(3,8)_ = 7.39, *P* = 0.011; Fig. 2]. In addition, the community composition of the cyanophage contigs was different in oxic sediment at station A compared to the hypoxic-anoxic sediment at stations D, E, and F (Bray-Curtis beta diversity, permutational multivariate analysis of variance [PERMANOVA], _pseudo_*F* = 5.71, *P* = 0.0006; Fig. 2). There was also a significant difference in Bray-Curtis beta diversity excluding the oxic station A, with the anoxic station F clustering differently from the stations D and E (PERMANOVA, _pseudo_*F* = 1.75, *P* = 0.03; Fig. S2). A similar pattern with higher alpha diversity and different community structure in the dead zone sediment was also observed when analyses were conducted on the classified taxonomy of the contigs (Fig. S3). These differences in cyanophage alpha and beta diversity are also visible in Fig. 2, and interestingly most cyanophage contigs with a high relative abundance in the oxic sediment at station A belonged to cluster x, while cyanophages in the hypoxic-anoxic sediment mainly belonged to clusters y and z (Fig. 3).

10.1128/mSphere.00208-21.2 FIG S2 NMDS showing Bray-Curtis beta diversity of the cyanophages between the stations excluding the oxic station A. The labels denote the station and each replicate number. Download FIG S2, PDF file, 0.1 MB. Copyright © 2021 Broman et al. 2021 Broman et al. https://creativecommons.org/licenses/by/4.0/ This content is distributed under the terms of the Creative Commons Attribution 4.0 International license.

10.1128/mSphere.00208-21.3 FIG S3 (A) Shannon’s H alpha diversity index based on the taxonomy of the cyanophage contigs (based on NCBI taxon identifiers). Contigs with the same classified taxon ID were merged for each sample (i.e., normalized count values summed). The one-way ANOVA was based on testing the whole model, with all four stations. (B) NMDS showing Bray-Curtis beta diversity of the cyanophage contig taxonomy (as described above) between the stations. The labels denote the station and each replicate number. Download FIG S3, PDF file, 0.2 MB. Copyright © 2021 Broman et al. 2021 Broman et al. https://creativecommons.org/licenses/by/4.0/ This content is distributed under the terms of the Creative Commons Attribution 4.0 International license.

### Cyanobacterial diversity and taxonomy.

**FIG 4:**
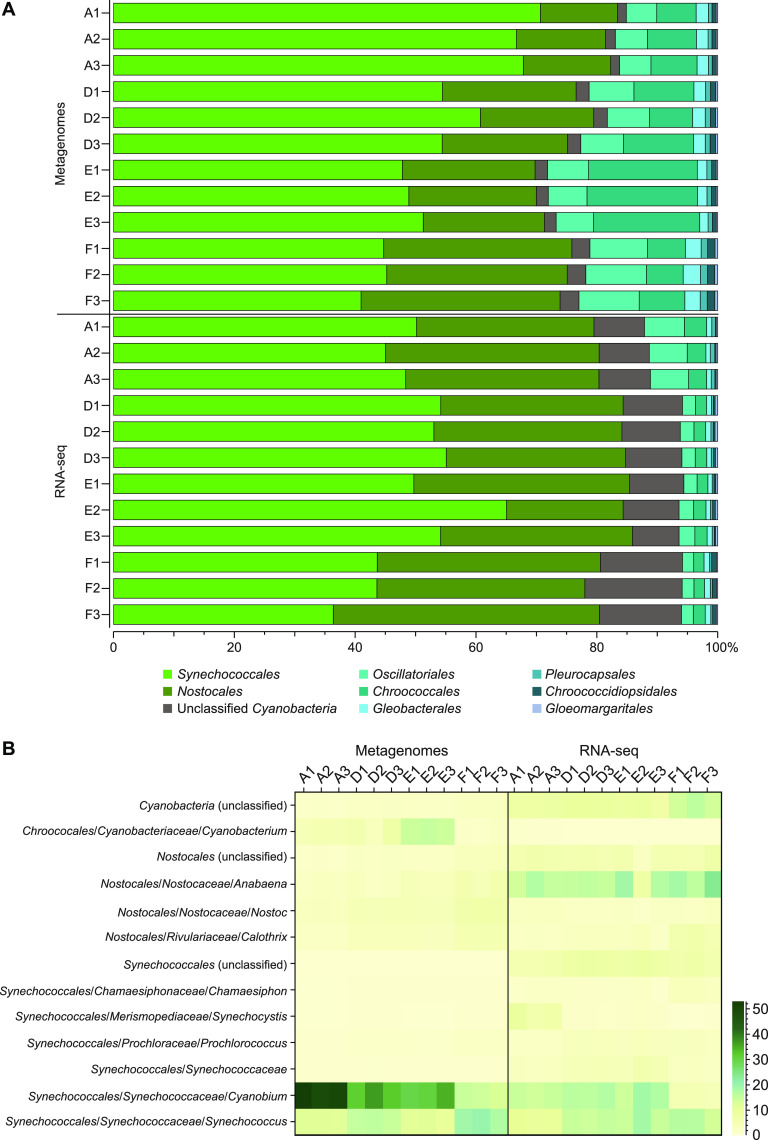
(A) The stacked bars show the relative proportion of classified DNA and RNA sequences against cyanobacterial genomes in the RefSeq database. The data show % of cyanobacteria, and the color legend shows taxonomic orders, while the *y* axis shows the triplicate samples for each station. (B) Heatmap showing the relative proportion of classified reads against cyanobacterial genomes in the RefSeq database. The data show the lowest taxonomic classification with at least an average of >2% in the DNA or RNA samples.

Cyanobacteria were detected in all metagenome and transcriptome sequencing (RNA-seq) sediment samples at all stations (*n* = 3 per station for both DNA and RNA data). The number of classified reads belonging to cyanobacteria was on average 3.3% (range 2.5 to 4.5%) in the DNA data and showed a decreasing trend along water depth (i.e., oxic station A to anoxic station F) from 7.9 to 4.2% (Fig. S4) in the RNA data. Both the cyanobacterial DNA and RNA data sets were dominated by populations belonging to the oxygenic photosynthetic class *Oxyphotobacteria*, with especially two taxonomic orders: (i) *Synechococcales* (up to 65% of the cyanobacteria in the RNA data) and (ii) *Nostocales* (up to 44% of the cyanobacteria in the RNA data; Fig. 4A). In the DNA data, there was an increasing pattern of reads classified to *Nostocales*, from 13% to 33% of all cyanobacteria, along the water depth gradient (i.e., stations A to F; Fig. 4A). Looking closer at the cyanobacteria in the hypoxic-anoxic sediment (stations D, E, and F), most of the classified reads were attributed to (i) the genera *Cyanobium* and *Synechococcus*, both belonging to the order *Synechococcales* (both in the DNA and RNA data), and (ii) the genus *Anabaena* belonging to the order *Nostocales* (15 to 20% of all cyanobacteria; mainly in the RNA data; Fig. 4B). Note that the reference database (NCBI RefSeq) might still label some species of *Dolichospermum* as *Anabaena*. *Cyanobium* was attributed more DNA and RNA-seq classified reads at the oxic station A than at the hypoxic-anoxic stations D, E, and F, with DNA 51% and RNA 14% of all cyanobacteria compared to DNA 14% and RNA 5%, respectively. *Synechococcus* showed the opposite pattern with higher numbers of reads at stations D, E, and F (DNA 19% and RNA 16% of all cyanobacteria compared to station A with DNA 12% and RNA 11%; Fig. 4B). These results indicate that cyanobacteria, even bloom-forming oxygenic photosynthetic taxa, are present and transcribe genes (as indicated in the mRNA data) in the dead zone sediment. A full list of read counts and sequence classifications against cyanobacterial genomes in the RefSeq database is available in Data Set S2.

10.1128/mSphere.00208-21.4 FIG S4 Proportion of metagenome and RNA-seq reads (%) classified against cyanobacterial genomes in the RefSeq database. The green color shows cyanobacteria, while the gray bars show the whole prokaryotic community. Download FIG S4, PDF file, 0.03 MB. Copyright © 2021 Broman et al. 2021 Broman et al. https://creativecommons.org/licenses/by/4.0/ This content is distributed under the terms of the Creative Commons Attribution 4.0 International license.

10.1128/mSphere.00208-21.6 DATA SET S2 Cyanobacterial results from the Kraken2 analysis against the NCBI RefSeq and taxonomy database. Download Data Set S2, XLSX file, 0.02 MB. Copyright © 2021 Broman et al. 2021 Broman et al. https://creativecommons.org/licenses/by/4.0/ This content is distributed under the terms of the Creative Commons Attribution 4.0 International license.

Cyanobacterial alpha diversity (Shannon’s H) in the sediment was significantly higher in the oxic station A (Shannon’s H 4.17 ± 0.02) than in the hypoxic-anoxic stations D, E, and F [Shannon’s H 3.92 to 3.94; one-way ANOVA, *F*_(3,8)_ = 129.76, *P* < 0.001; and Tukey *post hoc* tests *P* < 0.001].

### Cyanophages and associated cyanobacteria in the dead zone sediments.

**FIG 5:**
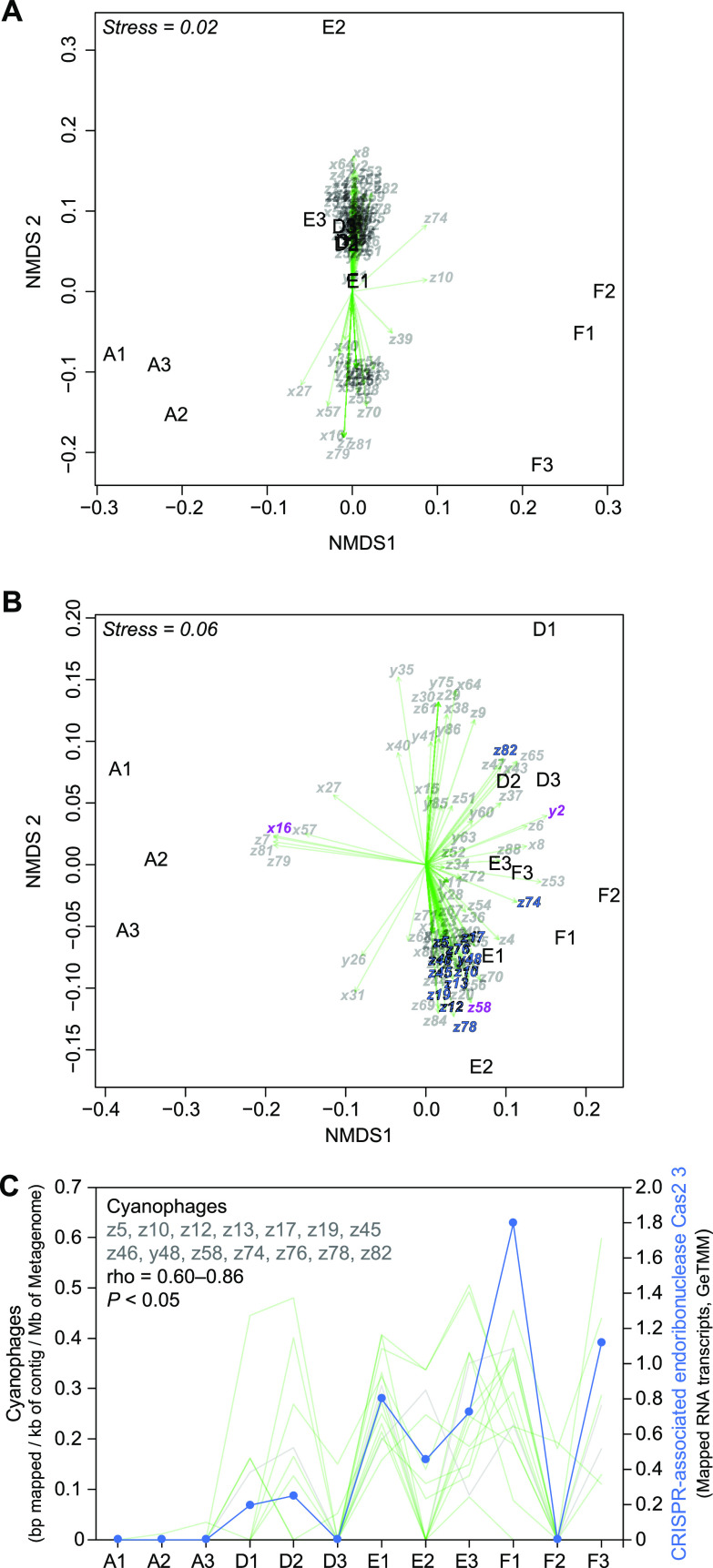
(A) NMDS showing the Bray-Curtis beta diversity based on the proportion of reads classified of the cyanobacterial RNA data (lowest taxonomic classification; stations shown on the graph). The overlying triplot shows the distribution of detected cyanophage contigs (light gray labels) in relation to the cyanobacterial beta diversity. The arrowheads denote the data point of the cyanophage. (B) NMDS showing the Bray-Curtis beta diversity of UniProtKB/Swiss-Prot-classified cyanobacterial proteins (mRNA data) identified in the data set (stations shown and denoted on the graph). Cyanophage contigs indicated by violet color (y2, x16, and z58) denote the cyanophages that together best explain the beta diversity of the cyanobacterial proteins (rho = 0.80). The blue cyanophage contigs (as well as violet z58) denote the cyanophage contigs in panel C that correlated with a cyanobacterium-related CRISPR protein. (C) Several of the cyanophage contigs in the hypoxic-anoxic sediment correlated positively with the number of mapped RNA transcripts against the metagenome assembly attributed to cyanobacterial protein CRISPR-associated endoribonuclease Cas2 3 (gene *cas2-3*). The light gray lines denote each cyanophage contig, while the blue line denotes the GeTMM values for the CRISPR protein.

The community compositions of the cyanophage contigs in the sediment were found to largely cluster with stations D and E. Similarly, the composition of cyanobacteria formed three clusters, stations D + E, A, and F, which were in relation to the oxygen concentrations at the stations (RNA data; Fig. 5A). The cyanophage contigs distributed in the sediments also showed an association with the cyanobacterial proteins. Here, cyanophage contigs x16, x27, x57, z7, z79, and z81 occurring at the oxic station A (Fig. 3) clustered toward the beta diversity of the UniProtKB/Swiss-Prot classified cyanobacterial proteins (mRNA data) in the sediment of station A (Fig. 5B). The majority of the cyanophage contigs and cyanobacterial proteins clustered with the hypoxic-anoxic stations (right side in Fig. 5B). BVSTEP analysis showed that the combination of the three cyanophage contigs y2, x16, and z58 best explained the beta diversity of the cyanobacterial proteins (rho = 0.80). That the cyanophages had an effect on the cyanobacterial community in the hypoxic-anoxic sediment was also indicated by several cyanophage contigs (z5, z10, z12, z13, z17, z19, z45, z46, y48, z58, z74, z76, z78, and z82) correlating positively with the number of mapped RNA transcripts attributed to cyanobacterial protein CRISPR-associated endoribonuclease Cas2 3 (gene *cas2-3*; rho = 0.60 to 0.86, *n* = 12 for each cyanophage, all *P* < 0.05; Fig. 5C). Finally, the taxonomy and protein classifications of the cyanophage contigs revealed that the majority were identified to genomes of *Synechococcus* or *Prochlorococcus* phages in the database (Data Sets S1 and S3) and were carrying a large number of gene copies related to hypothetical proteins but also, e.g., glycosyl transferase-related genes, heat shock protein IbpA, p-starvation-inducible protein (gene *phoH*), and photosystem II D1 (*psbA* genes) (Data Set S3).

10.1128/mSphere.00208-21.7 DATA SET S3 BLASTP results of classified cyanophage ORFs. Download Data Set S3, XLSX file, 0.08 MB. Copyright © 2021 Broman et al. 2021 Broman et al. https://creativecommons.org/licenses/by/4.0/ This content is distributed under the terms of the Creative Commons Attribution 4.0 International license.

### Cyanobacterial RNA transcripts in the dead zone sediments.

**FIG 6:**
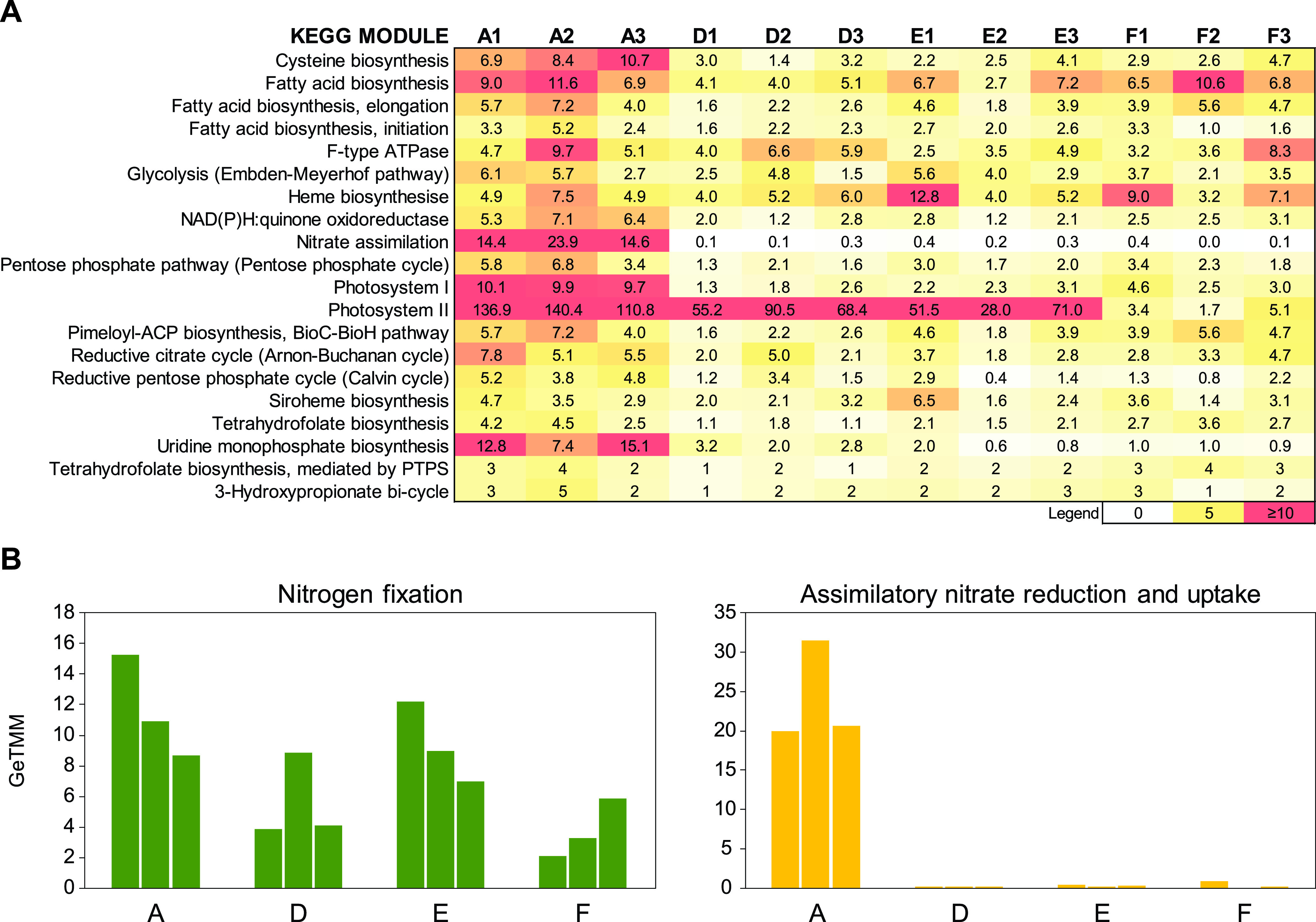
(A) Cyanobacterial RNA transcripts classified against the UniProtKB/Swiss-Prot linked to KEGG modules. The heatmap shows the top 20 modules and normalized sequence counts (GeTMM). Note that proteins might be associated with more than one module. (B) Cyanobacterial RNA transcripts attributed to “N_2_ fixation” and “assimilatory nitrate reduction and uptake” based on UniProtKB/Swiss-Prot classifications. Note the different *y* axes between subpanels.

The number of cyanobacterial RNA transcripts was significantly higher at station A (363 ± 45 GeTMM [*ge*ne length corrected *t*rimmed *m*ean of *M*-values]) compared to the hypoxic-anoxic stations D, E, and F (170 ± 37, 158 ± 42, and 126 ± 21, respectively; *F* = 24.9, *P* < 0.001, one-way ANOVA with Tukey *post hoc* test; Fig. 6A). RNA transcripts for the KEGG module glycolysis were prevalent at all stations, indicating that anaerobic carbon metabolism was likely occurring. Furthermore, RNA transcripts attributed to other KEGG modules present at all stations included, e.g., fatty acid biosynthesis, proteins that were part of the reductive citrate cycle (Arnon-Buchanan cycle), cystine amino acid synthesis, and the photosystems (Fig. 6A). Photosystem I had more RNA transcripts at station A (*F* = 85.7, *P* < 0.001), while the photosystem II had a different number of RNA transcripts for each station (when each station was tested against the others [*F* = 31.3, *P* < 0.05; Fig. 6A]). The number of RNA transcripts attributed to nitrate assimilation was significantly higher at station A than at all other stations (*F* = 30.8, *P* < 0.001; Fig. 6A). Because not all proteins could be classified into KEGG modules, we also decided to inspect the UniProtKB/Swiss-Prot-classified protein data manually. A full list of proteins affiliated with cyanobacteria, KEGG KOs plus modules, and a list of proteins with a significant difference between stations are available in Data Set S4.

10.1128/mSphere.00208-21.8 DATA SET S4 (1) The first sheet shows GeTMM values for proteins classified to reference cyanobacterial species in the UniProtKB/Swiss-Prot database. (2) The second sheet shows GeTMM values for proteins and their KEGG KO and KEGG modules classifications. (3) The third sheet shows the results of the edgeR analysis. Only statistically significant proteins are shown (FDR < 0.05). The table shows logFC (fold change) values for pairwise comparisons (sample 1 versus sample 2), and values of >0 denote more RNA transcripts in sample 1. Values higher in sample 1 are denoted by red cells, while values higher in sample 2 are denoted by green cells. Note that there were no differences between stations D and E. Download Data Set S4, XLSX file, 1.4 MB. Copyright © 2021 Broman et al. 2021 Broman et al. https://creativecommons.org/licenses/by/4.0/ This content is distributed under the terms of the Creative Commons Attribution 4.0 International license.

RNA polymerase was present at all stations in the cyanobacterial RNA data (Data Set S4); also, the oxic station A had more RNA transcripts classified to ribosomal proteins (30S and 50S) than did the other stations under hypoxic and anoxic conditions (edgeR analysis, false-discovery rate [FDR] < 0.05). Furthermore, the number of significantly different proteins between station A and stations D, E, and F increased with water depth, with A versus D, 58 proteins; A versus E, 69 proteins; and A versus F, 90 proteins (Data Set S4). This shows that with decreasing oxygen concentrations, and increasing distance from oxic station A, the difference in cyanobacterial metabolism increases.

Cyanobacterial RNA transcripts for N_2_ fixation (genes *nifDEHKNU*) were present at all stations (Fig. 6B). The number of *nifU* RNA transcripts was higher at station A than at D and F (FDR < 0.05), and *nifH* RNA transcripts were higher at station E than at station A (FDR < 0.05). Genes related to assimilatory nitrate reduction and uptake (nitrate reductase *narB* and nitrate transporters *nrtABCDP*) had together more RNA transcripts at the oxic station A than at the hypoxic-anoxic stations (20 to 30 GeTMM at station A compared to <1 GeTMM at the other stations; Fig. 6B). Of these, RNA transcripts attributed to genes *narB* and *nrtP* were significantly higher at station A (FDR < 0.05) than at stations D, E, and F (Data Set S4).

## DISCUSSION

We detected cyanophages at all four stations along the oxic-anoxic gradient. Viruses in sediment are affected by water flow and environmental shifts due to seasonal changes, as well as microbial abundances and activity (35). However, dead zones in the Baltic Sea are relatively stable environments due to a strong halocline trapping the heavy, more saline and oxygen-deficient bottom water (23). In the sampled gradient, we detected that cyanophages (based on partial genome contigs and classified closest relatives according to NCBI NT) had a higher alpha diversity and different beta diversity in the hypoxic-anoxic sediment compared to the oxic station A, which confirmed our first hypothesis. This might partly be explained by a higher abundance of viruses in deep anoxic water, which has been reported in the Baltic Sea (36) and in other anoxic areas such as the Cariaco basin in the Caribbean Sea (37). In addition, cyanophages were indicated to have an association with different cyanobacterial communities and their RNA transcripts across the sampled gradient (as indicated in Fig. 5A and B), confirming our second hypothesis. This indicates that cyanobacteria in the hypoxic-anoxic sediment have to cope with a more diverse cyanophage community compared to that of the oxic sediments. Considering that cyanophages can persist for thousands of years in anoxic sediments (31), differences in the cyanophage community composition and diversity might also be an effect of accumulating viruses in dead zone sediments. Cyanobacterial CRISPR-associated endoribonuclease-related RNA transcripts (attributed to gene *cas2-3*) were correlated with the relative abundance of several cyanophages, possibly due to viral infection. On the bacterial genome, CRISPR (clustered regularly interspaced short palindromic repeats) loci consist of virus-specific DNA repeats with adjacent *cas* genes. Upon viral infection, the virus DNA is sampled and inserted into a CRISPR locus and transcribed into crRNA, which is then used to guide Cas endonuclease proteins to cleave invading virus DNA (38). Our findings suggest that cyanobacteria were targeted by cyanophages in the sampled sediments. However, we were unable to retrieve any counts after mapping the RNA data to the cyanophage genes, likely due to (i) insufficient mRNA reads, considering that any viral transcription would be very small compared to the whole RNA data set, and/or that (ii) a substantial portion of the cyanophages were present without infected hosts. It has previously been shown that cyanophages can persist for centuries in the deep anoxic Baltic Sea (37 m below the seafloor) (31), and it is therefore possible that a large portion of cyanophages were present in our sampled sediments but not infecting any host cells. Considering that our data indicate that cyanobacteria were transcribing genes in all the sampled stations, it is possible that at least some cyanophages were infecting host cells.

Viral lysis of cyanobacterial cells contributes carbon, phosphorus, and nitrogen to other microorganisms (5, 6). The bacterial uptake of lysed material has been shown to be especially important in oligotrophic environments where nutrients are scarce (39), and our results suggest that the presence of cyanophages in dead zone sediments could result in cyanobacterial lysed cells and support elevated nutrient turnover. This phage-driven lysis of cyanobacteria and consequence for nutrient turnover would, nevertheless, need to be confirmed in future studies. Cyanophages are known to carry genes for the photosystem II (40), as confirmed by our results. However, in contrast to cyanophages that were limited to *psbA* genes, the RNA transcript data for cyanobacteria were attributed to multiple different photosynthesis-related genes (at least 30 genes detected in this study). In more detail, the cyanobacterial photosynthesis data set was dominated by *psbA* genes at all stations except the anoxic station F. It is therefore possible that changes in cyanobacterial photosynthesis regulation could have partly been due to viral infection by cyanophages. Our results indicate that prokaryote-virus interactions are an important factor to consider in future studies looking at prokaryotic photosystem genes or protein counts. Similarly, cyanophages were also found to carry genes for the phosphorus regulon *phoH*. Even though the function of the translated protein is still not fully understood (40), the transcription of the *phoH* gene has been shown to be induced by phosphate starvation (41). Cyanophages might therefore have influenced the cycling of phosphate by infecting cyanobacteria in the sediment. Further studies are needed to investigate the impact of such nutrient fluxes in dark and anoxic sediments. We were not able to determine if cyanophages influenced the community composition of the cyanobacteria (or vice versa), or how many of the assembled partial cyanophage genomes in this study were derived from sinking pelagic cyanobacteria or were already residing in the sediment. However, the difference in cyanophage alpha and beta diversity between the oxic and hypoxic-anoxic sediment suggests that (i) host-associated cyanobacteria capable of surviving in oxygen-deficient environments select for specific cyanophages and/or (ii) cyanophages in dead zones might have a lower decay rate (36) and be more tolerant to the certain environmental conditions, such as H_2_S, which has been shown to have antiviral properties for respiratory viruses (42).

In addition to the cyanophages, our DNA and RNA data also show that cyanobacteria were present in the dead zone sediments. This is in agreement with previous studies showing that cyanobacteria are able to survive in dark and anoxic environments either as akinetes (24) or by utilizing fermentative metabolism (43). Furthermore, cyanobacterial RNA transcripts related to RNA processing and modification, protein biosynthesis, protein processing, and cyanobacterial cold shock protein have been detected *in situ* in the anoxic sediment at the deepest point in the Baltic Sea (Landsort deep, 466 m) (44). The presence of the filamentous genus *Anabaena* (potentially containing planktonic species belonging to the renamed *Dolichospermum* genus, and/or as benthic *Anabaena*) in our study indicated that akinetes or fermentative metabolism could have been possible. Interestingly, the relative proportion of classified reads attributed to *Anabaena* was higher in the RNA-seq than in the DNA data, and this might be explained by the following: (i) *Anabaena* is known to produce akinetes (24) and (ii) certain species of *Anabaena* are known to be heterotrophic and survive in darkness even under anaerobic conditions (43). The presence of *Anabaena* in the hypoxic-anoxic sediment could also be related to the lower rate of organic matter degradation in oxygen-deficient sediments (45, 46), resulting in pelagic cyanobacterial material being likely preserved for a considerable time. However, RNA transcript data directly affiliated with cyanobacteria suggest that at least some of these organisms are actively transcribing genes in dead zone sediments. *Anabaena* might use fermentative metabolism (endo- or exogenous [43]) or akinete resting stages (with a low metabolic signal that was present in the RNA data). For example, the species Anabaena cylindrica has been observed to produce H_2_ by utilizing endogenous substrates (47). In contrast to *Anabaena*, the other top genera detected in the hypoxic-anoxic sediment *Cyanobium* and *Synechococcus* are not known to form akinetes (48). However, *Synechococcus* has been shown to survive in anoxic and dark waters in the Black Sea with strong indications of being capable of fermentation (49). Our mRNA data further suggest that these cyanobacteria are present in dark and anoxic environments.

The mRNA data from cyanobacteria in the hypoxic-anoxic sediment also indicate that N_2_-fixation genes are being transcribed by cyanobacteria in dead zones. We detected the highest number of N_2_-fixation-related RNA transcripts in the oxic station A, possibly due to there being a higher relative abundance of cyanobacteria at that station than at the hypoxic-anoxic stations. The sampled hypoxic-anoxic stations have low or no nitrate and nitrite (50), which explains why assimilatory nitrate reduction and uptake were prevalent only in the oxic sediment at station A. That N_2_ fixation was possible in the dead zone sediment was indicated by the presence of *Dolichospermum*/*Anabaena* known to carry heterocysts to conduct N_2_ fixation (7). Furthermore, non-heterocystous cyanobacteria such as certain *Synechococcus* species have also been described to conduct N_2_ fixation (51). However, Baltic Sea dead zone sediments have been shown to be rich in ammonium (50), which is a favorable bioavailable source of nitrogen for some cyanobacteria (52). It is therefore possible that N_2_ fixation was conducted inside specific microniches in the heterogenous sediment (such as biofilms or on/inside particles) where ammonium might have been scarce. However, further controlled studies are needed to investigate if cyanobacterial N_2_ fixation is an important metabolic process in dead zone sediments. We were also able to detect RNA transcripts classified to genes coding for photosynthesis at all sampled stations. The highest number of photosynthesis RNA transcripts was detected at the oxic station A, possibly due to cyanobacteria having the highest relative abundance at that station. Accumulation of RNA transcripts attributed to the photosystem in darkness has previously been observed for the strain *Synechocystis* sp. strain PCC 6803 (53), and an increase in photosystem II units for Anabaena variabilis ATCC 2941 (25). Similarly to previous laboratory studies mentioned above, our data also indicate that heterotrophic cyanobacteria in dark anoxic sediments produce photosynthesis-related RNA transcripts.

We have here shown that cyanophages and cyanobacteria are present in one of the largest dead zones in the world. Cyanophages were detected at all stations and had a different beta diversity in the oxic sediment than in the hypoxic and anoxic sediments, suggesting that cyanobacteria and/or the environment select for specific cyanophages. Moreover, our results show that these cyanophages can infect cyanobacteria, affecting the photosystem and phosphate regulation. Top prevalent cyanobacteria detected include genera not capable of forming akinetes (*Cyanobium* and *Synechococcus*) and akinete-forming *Dolichospermum*/*Anabaena*. Cyanobacterial RNA transcripts in sediment were classified to, e.g., anaerobic glycolysis and N_2_ fixation. Considering that large amounts of cyanobacteria sinking to dead zone sediments are known to fuel the benthic ecosystems with phosphorus (so-called internal loading), our cyanophage data indicate the potential for viral lysis of cyanobacteria, and this might sustain the high nutrient turnover in these heavily eutrophied environments.

## MATERIALS AND METHODS

### Study area and sampling.

Sediment was collected using a modified box corer (54) during 23 to 26 April 2018 from four stations, located below the euphotic zone, following a water depth and oxygen gradient (stations A, D, E, and F) in the dead zone of Eastern Gotland basin, Baltic Sea (the stations and geochemical data have previously been presented in the work of Marzocchi et al. [55]) (Table 1; 2018 oxygen data first presented in the work of Broman et al. [33]). Three polyvinyl chloride (PVC) cylinders (5-cm diameter; 30-cm length) from each site were inserted into the box-core sediment and moved onto a sterile bench. The sediment was gently extruded, and the top 0 to 2 cm directly sliced into a 50-ml centrifuge tube. The sample was directly flash frozen and kept in liquid N_2_ until being transferred and stored at −80°C.

### Nucleic acid extraction.

DNA and RNA were extracted from thawed and homogenized sediment with the DNeasy PowerMax soil kit (Qiagen) and RNeasy PowerSoil kit (Qiagen), with 10 g and 2 g input material, respectively. Extracted RNA was DNase treated with the Turbo DNA-free kit (Invitrogen) and rRNA depletion using the RiboMinus transcriptome isolation kit (bacterial version, ThermoFisher Scientific). Multiplexed libraries were prepared with the ThruPLEX DNA-seq (Rubicon Genomics) and TruSeq RNA Library Prep v2 [Illumina, without the poly(A) selection step] kits for DNA and RNA, respectively. Paired-end 2- by 150-bp sequencing was conducted at the Science for Life Laboratory, Stockholm, Sweden, on the Illumina NovaSeq platform (DNA on one lane, NovaSeq6000 S2, and RNA on one lane, Illumina NovaSeq6000 S4). The sequencing yielded on average 41 million (range 32 to 52) and 82 million (range 74 to 89) read-pairs for each DNA and RNA sample, respectively.

### Bioinformatics.

Illumina adapters were removed from the raw sequence data by using SeqPrep 1.2 (56) on default settings targeting the adapter sequences. Phi-X174 control sequences were removed from the data by mapping the reads with bowtie2 2.3.4.3 (57) against the PhiX genome (NCBI reference sequence: NC_001422.1). Reads were quality trimmed using Trimmomatic 0.36 (58) with the parameters LEADING:20 TRAILING:20 MINLEN:50, yielding quality-trimmed reads with an average read length of 137 and 140 bp for the DNA and RNA data, respectively.

Metagenome and RNA-seq quality-trimmed sequences (Trimmomatic: paired without unpaired, PwU) were classified against the NCBI RefSeq genome database (downloaded 1 March 2019) using Kraken2 2.0.7 with a paired-end setup (59). The DNA data consisted on average of 103,043 reads mapped to cyanobacterial genes (range 51,939 to 152,036), while the RNA data consisted on average of 3,860,948 reads mapped to cyanobacterial genes (range 2,647,420 to 5,345,457). The RNA samples still contained on average 89% rRNA sequences (range 87 to 92%) after ribosomal depletion and therefore contained a large amount of rRNA alongside mRNA, and this likely helped to further identify the cyanobacterial community in the sediment. This method made it possible to classify a large amount of reads and detect cyanobacteria. However, because the reads classify against genes with different lengths in the RefSeq genomes, it is not possible to directly compare minor differences in relative abundances of taxonomic groups (although it gives a good indication of the distribution of reads). The final taxonomy data were analyzed and visualized as a relative proportion of classified reads (%) in the software Explicet 2.10.5 (60).

The DNA PwU quality-trimmed reads were used to construct a metagenome coassembly using MEGAHIT 1.1.2 on default settings (61). The Prokka 1.12 software tool (62) was used for gene prediction with Prodigal 2.6.3 (63) and annotation using BLAST 2.6.0+ (64) against the UniProtKB/Swiss-Prot database (database downloaded 31 January 2019) (65), with the parameters ––proteins uniprot_sprot.fasta ––metagenome. DNA and RNA PwU quality-trimmed reads were then mapped onto the coassembly using bowtie2 on default settings, and the output .sam files were converted to .bam with SAMtools 1.9 (66). Final sequence counts were estimated by using HTSeq-count from the HTSeq python package 0.9.1 (67) with the .bam files and PROKKA output .gff file as input, with the parameters -s no -f bam -t CDS -i ID. Sequence counts were normalized among samples as *ge*ne length corrected *t*rimmed *m*ean of *M*-values (GeTMM) (68). Unique UniProtKB/Swiss-Prot identifiers were merged, and identifiers affiliated with cyanobacteria in the UniProtKB/Swiss-Prot database were extracted. The final data consisted on average of 175,134 mapped cyanobacterial DNA sequences per sample (range 143,600 to 209,247) and an average of 5,080 mapped cyanobacterial RNA transcripts per sample (range 4,075 to 6,048). Proteins were categorized into KEGG modules (69) by linking UniProtKB/Swiss-Prot identifiers to KEGG KO identifiers using the “Retrieve/ID mapping” function available on the official UniProt website, https://www.uniprot.org/uploadlists/.

Contigs longer than 2,000 bp in the metagenome coassembly were extracted using sqtek 1.3 and identified as viruses with VirSorter 1.0.3 (70) using the supplied RefseqABVir database on default settings via the CyVerse Discovery Environment interface (https://de.cyverse.org). Contigs classified as “Pretty sure” and “Quite sure” by VirSorter were used for further analyses. The contigs were classified for taxonomy using BLAST 2.10.1+ (64) against the NCBI NT database (data: 14 January 2021) with the following parameters: -max_target_seqs 5 -outfmt 6 -evalue 0.001 -task blastn. The first listed hit with the highest bit score was used for each contig. Open reading frames (ORFs) were detected in these contigs by using prodigal 2.6.3 on default settings (63). To predict potential cyanophages, network analysis with isolated genome-sequenced bacterial and archaeal viruses was conducted using vConTACT2 v0.9.8 with default settings (NCBI Bacterial and Archaeal Viral RefSeq V88 database [34]). vConTACT uses the Markov Cluster Algorithm (MCL) with an inflation value of 2 to cluster similar protein sequences (34). The ORFs were further classified using BLASTP 2.9.0+ against a database of all viral genomes from the NCBI RefSeq database (downloaded date: 12 July 2019) with the parameters -max_target_seqs 1 -outfmt 6 -evalue 0.001 to verify the cyanophages. Bowtie2 was used to map DNA PwU reads on the contigs and ORFs with a 90% identity alignment threshold. Considering the reads had an average length of 137 bp after quality trimming, using Bowtie2 parameters “–score-min L,0,-0.6.” allows for 14 mismatches without including additional gap penalties. The output .sam files were converted to .bam and sorted by coordinates using SAMtools 1.9 (66). BEDTools 2.27.1 (71) was used to calculate total read depth per contig (mapped bp) and coverage per contig (%) using parameters “bedtools genomecov -d” and “bedtools genomecov -pc.”, respectively. SAMtools idxstats were used to retrieve mapped counts of cyanophage ORFs for the DNA samples (no RNA reads could be mapped to the cyanophage ORFs). The data set was then delimited to cyanophage-identified contigs with a coverage of at least 75% to ensure the viral genome was present in the sample, following the protocols of previous ocean viral surveys (72). The amount of “mapped bp per contig” was normalized among samples by first dividing the mapped bp for each contig with the length of the contig (kb), followed by dividing with the total number of bases in the metagenome (Mb) (72). A full list of bioinformatic statistics is available in Data Set S5 in the supplemental material, such as number of reads obtained after sequencing, quality trimming, and mapping against the metagenome coassembly and numbers of reads classified against the UniProtKB/Swiss-Prot database.

10.1128/mSphere.00208-21.9 DATA SET S5 Number of reads obtained after sequencing, quality trimming, classification for cyanobacterial taxonomy against the NCBI RefSeq database, mapping against the metagenome coassembly, and classification for cyanobacterial proteins against the UniProtKB/Swiss-Prot database. Download Data Set S5, XLSX file, 0.01 MB. Copyright © 2021 Broman et al. 2021 Broman et al. https://creativecommons.org/licenses/by/4.0/ This content is distributed under the terms of the Creative Commons Attribution 4.0 International license.

### Statistics.

Shannon’s H alpha diversity was calculated on the normalized sequence counts for the cyanophage contigs as well as their classified NCBI NT taxonomy (by summing counts with the same contig NCBI taxon identifier for each sample) using the software PAST 3.25 (73). Shannon’s H alpha diversity index for the cyanobacteria was calculated in the software Explicet based on subsampling counts to the lowest sample size (2,647,420 counts) and bootstrap × 100 (with the mean reported as the final alpha index). Nonmetric multidimensional scaling (NMDS) showing Bray-Curtis beta diversity was based on the normalized counts for the cyanophage contigs and analyzed in the software PAST 3.25. NMDS plots of cyanobacterial community structure or cyanobacterial protein beta diversity with cyanophages as overlying triplot were constructed using the metaMDS function in the vegan R package (74). The best explanatory cyanophage for the cyanobacterial community structure was analyzed with the BVSTEP method (75) using R 3.6.0 (76) and the bvStep function with default settings in the sinkr R package (77). The bvStep was run by using the distance methods Bray-Curtis for cyanobacterial proteins and Euclidean distances for cyanophages. Spearman correlations were conducted to find patterns between cyanophage contigs and cyanobacterial proteins by using the function rcorr in the Hmisc R package (78). Differences in alpha diversity between stations were tested with one-way ANOVAs and *post hoc* Tukey tests using IBM SPSS 26. Differences in the beta diversity between stations were tested with PERMANOVA (9,999 permutations) using the software PAST. Differences in RNA transcripts for annotated proteins between stations were tested with the R package edgeR 3.24.3 (79) by using the “run_DE_analysis.pl” script supplied with Trinity 2.8.2 (80). False-discovery rates (FDR) of <0.05 were used to indicate statistical significances.

### Data availability.

The raw sequencing data supporting the conclusions of this article are available in the NCBI BioProject repository, PRJNA531756 (81).
