# Supplementary figures and images for: Cyanophage Diversity and Community Structure in Dead Zone Sediments
Source: mSphere. 2021 Apr 28;6(2):e00208-21. doi: 10.1128/mSphere.00208-21 (PMC8092138; doi:10.1128/mSphere.00208-21)

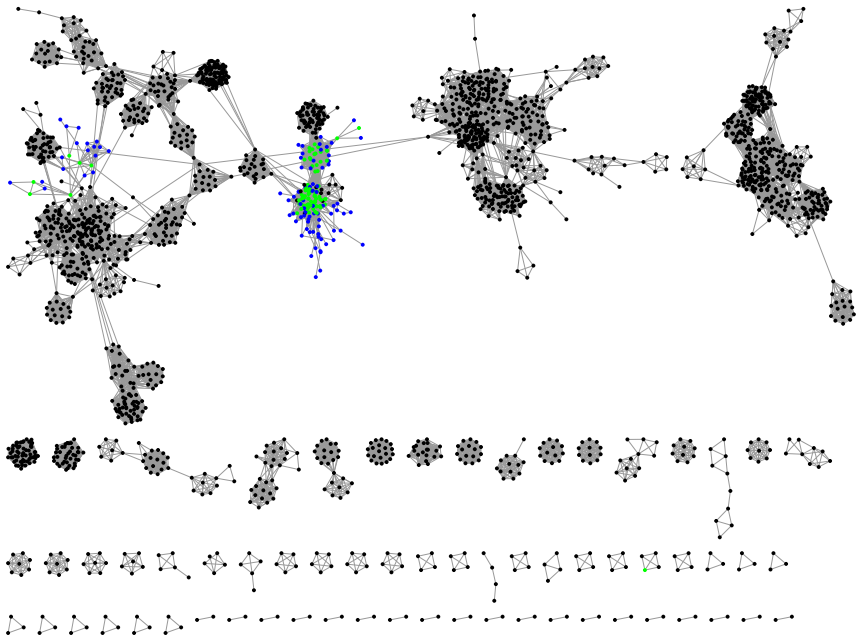

Supplement: FIG S1 [file mSphere.00208-21-sf001.pdf]

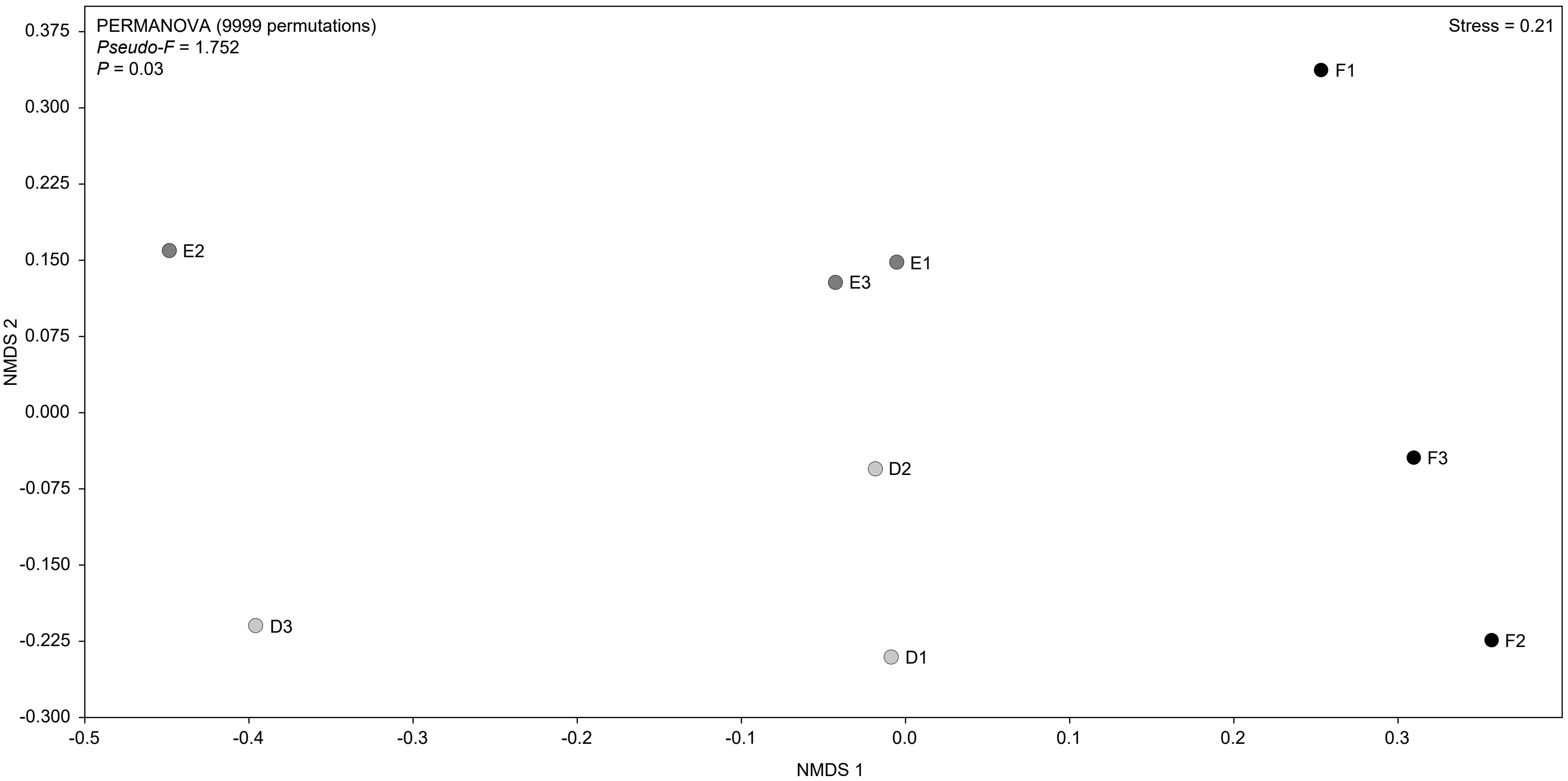

Supplement: FIG S2 [file mSphere.00208-21-sf002.pdf]

**A**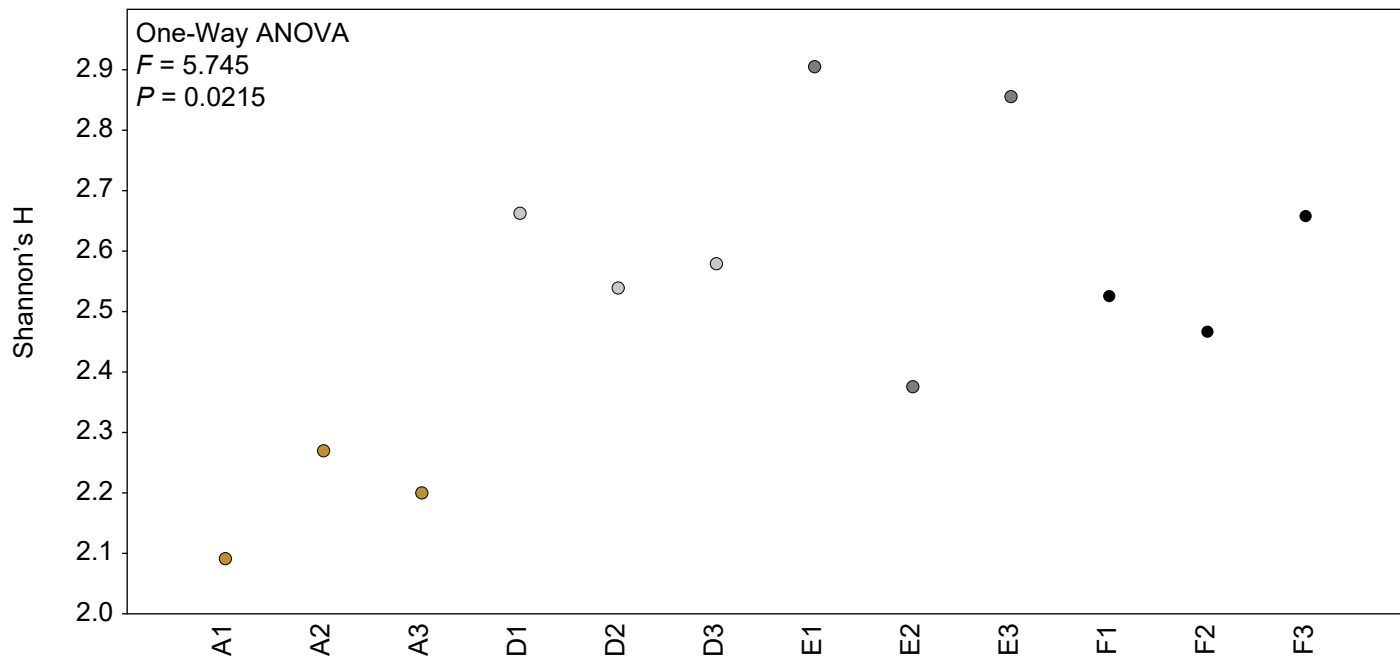**B**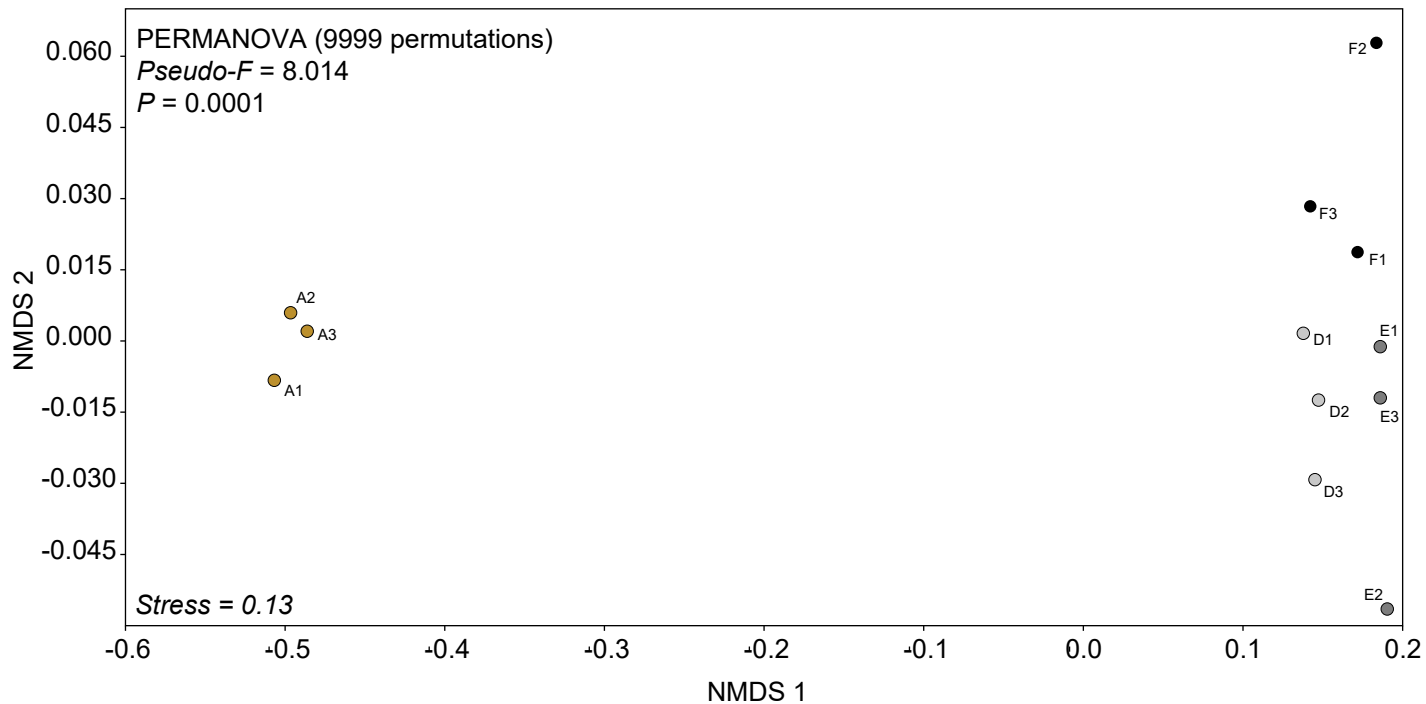

Supplement: FIG S3 [file mSphere.00208-21-sf003.pdf]

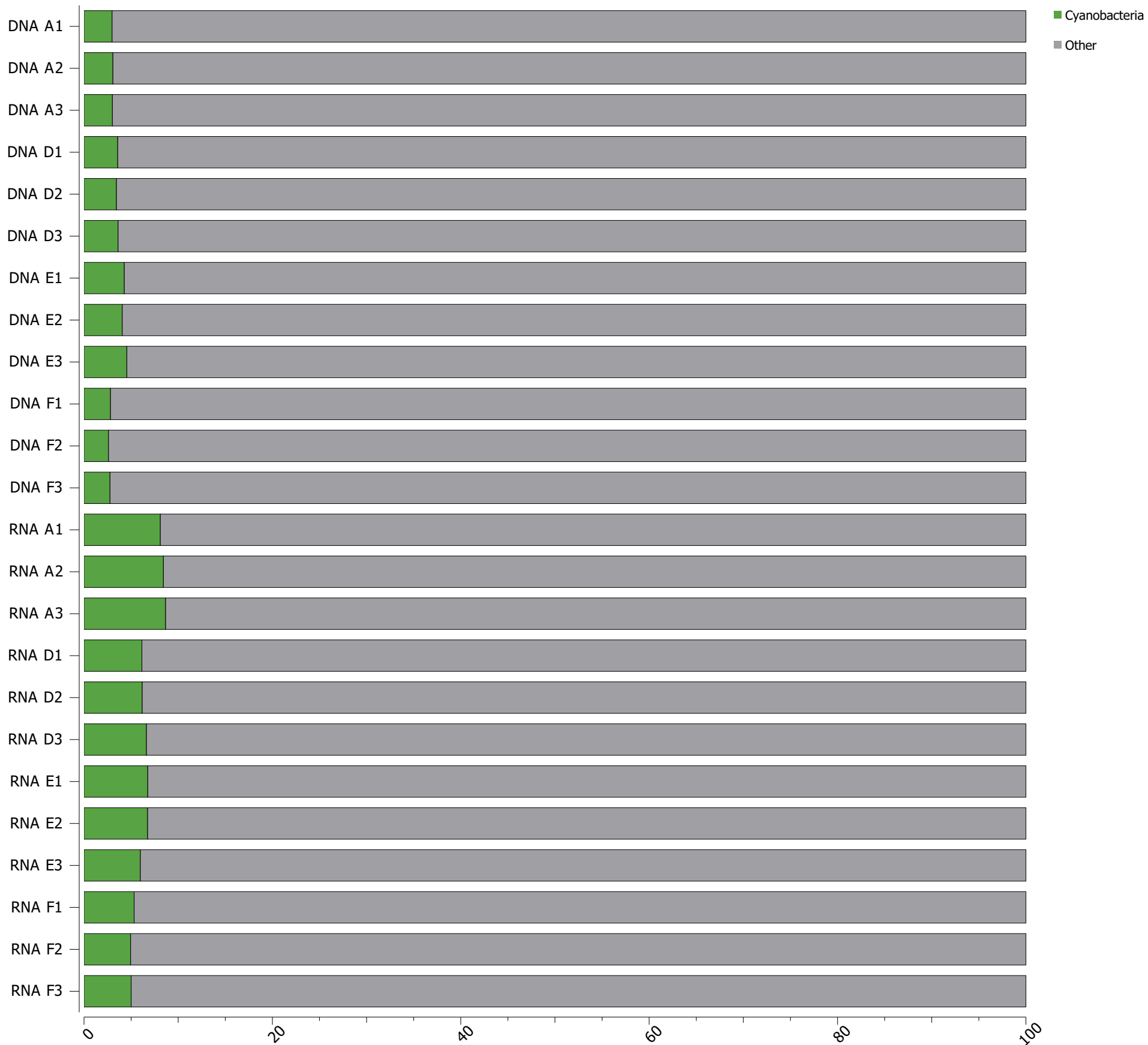

Supplement: FIG S4 [file mSphere.00208-21-sf004.pdf]
